# Binding of Macrophage Receptor MARCO, LDL, and LDLR to Disease-Associated Crystalline Structures

**DOI:** 10.3389/fimmu.2020.596103

**Published:** 2020-12-08

**Authors:** Anika Alberts, Annika Klingberg, Leonie Hoffmeister, Anne Kathrin Wessig, Korbinian Brand, Andreas Pich, Konstantin Neumann

**Affiliations:** ^1^ Institute of Clinical Chemistry, Hannover Medical School, Hannover, Germany; ^2^ Research Core Unit Proteomics & Institute of Toxicology, Hannover Medical School, Hannover, Germany

**Keywords:** sterile inflammation, gout, urate, uric acid, crystallopathies, inflammasome, atherosclerosis

## Abstract

Endogenous and exogenous crystalline structures are involved in various pathologies and diseases in humans by inducing sterile inflammation, mechanical stress, or obstruction of excretory organs. The best studied of these diseases is gout, in which crystallization of uric acid in the form of monosodium urate (MSU) mainly in synovial fluid of the joints leads to sterile inflammation. Though some of these diseases have been described for centuries, little is known about if and how the immune system recognizes the associated crystals. Thus, in this study we aimed at identifying possible recognition molecules of MSU using liquid chromatography-mass spectrometry (LC-MS) analysis of MSU-binding serum proteins. Among the strongest binding proteins, we unexpectedly found two transmembrane receptors, namely macrophage receptor with collagenous structure (MARCO) and low-density lipoprotein (LDL) receptor (LDLR). We show that recombinant versions of both human and mouse MARCO directly bind to unopsonized MSU and several other disease-associated crystals. Recombinant LDLR binds many types of crystals mainly when opsonized with serum proteins. We show that this interaction is predominantly mediated by LDL, which we found to bind to all crystalline structures tested except for cholesterol crystals. However, murine macrophages lacking LDLR expression do neither show altered phagocytosis nor interleukin-1β (IL-1β) production in response to opsonized crystals. Binding of LDL to MSU has previously been shown to inhibit the production of reactive oxygen species (ROS) by human neutrophils. We extend these findings and show that LDL inhibits neutrophil ROS production in response to most crystals tested, even cholesterol crystals. The inhibition of neutrophil ROS production only partly correlated with the inhibition of IL-1β production by peripheral blood mononuclear cells (PBMCs): LDL inhibited IL-1β production in response to large MSU crystals, but not small MSU or silica crystals. This may suggest distinct upstream signals for IL-1β production depending on the size or the shape of the crystals. Together, we identify MARCO and LDLR as potential crystal recognition receptors, and show that LDL binding to diverse disease-associated crystalline structures has variable effects on crystal-induced innate immune cell activation.

## Introduction

Crystallopathies are a diverse set of medical conditions where the formation of crystals is the basis of or at least a contributing factor to the disease (1, 2). Deposition of monosodium urate (MSU) crystals leads to gout (3) and formation of calcium pyrophosphate dihydrate (CPPD) induces pseudogout (4), while the accumulation of cholesterol crystals in blood vessel walls contributes to atherosclerosis development. Human kidney stones mostly consist of calcium oxalate crystals. Moreover, inhalation of environmental particles, e.g., silica or asbestos, causes long-term inflammation and scarring of the lung (5). Vaccine adjuvants often contain alum salts or alhydrogel which may also enhance immune responses partially due to its crystalline nature.

Crystalline structures induce inflammation in various ways: MSU crystals cause activation of neutrophils (6) leading to formation of neutrophil extracellular traps (NETs) (7, 8) and activation of the complement system (9, 10). Monocytes respond to MSU crystals with strong pro-inflammatory cytokine production such as interleukin-1β (IL-1β), IL-6, or tumor necrosis factor-α (TNF-α) (11–13), while macrophages require a priming signal e.g., C5a binding (14). Secretion of mature IL-1β requires crystal-induced activation of the NLRP3 inflammasome (15). Moreover, recent studies suggested necroptosis (a programmed form of necrosis/inflammatory cell death) as another molecular mechanism behind the crystal-induced inflammation (16).

Most studies on the recognition mechanisms of crystalline structures have focused on MSU crystals. The receptors CD11b, CD16, and CD14 have been shown before to be involved in activation of immune cells by MSU crystals, but it is unclear if they directly bind to crystalline structures (17, 18). Apolipoproteins ApoB and ApoE bind to MSU crystals and inhibit neutrophil activation by these crystals (19–21). Environmental particles like titanium dioxide (TiO_2_) have been described to be bound and phagocytosed by macrophage receptor with collagenous structure (MARCO, also known as SR-A6 or SCARA2) (22), which is important in models of silicosis (23). In a previous study, we identified C-type lectin domain family 12 member A (Clec12A; also known as MICL and CLL-1) as a specific inhibitory receptor for MSU crystals (24). Moreover, we found the acute phase protein C-reactive protein (CRP) to recognize MSU crystals and to recruit proteins of the complement cascade to the crystal surface (C1 and MASP-1) (25).

These data suggest that the immune system may have developed further specific recognition molecules for crystalline structures, which—like CRP—may recognize microbes, damaged host cells, and crystal surfaces. Here, we continued our search for specific crystal recognition molecules in human body fluids using mass spectrometry. Unexpectedly, we found a transmembrane receptor that directly binds to naked crystals and a transmembrane receptor that indirectly binds to opsonized crystalline structures.

## Materials and Methods

If not stated otherwise, all plastic materials were purchased from Sarstedt, Germany.

### Human Body Fluids and Isolation of Immune Cells

Normal human serum was obtained by drawing venous blood from healthy donors (CRP concentration 0.3–2.4 µg/ml) into clotting activator-coated blood collection tubes (Sarstedt, #02.1388). Following incubation for 30 min at 4°C, it was collected by centrifugation at 3000 xg for 10 min at room temperature (RT) and stored at −80°C. As an acute phase reaction serum, a leftover diagnostic serum sample of a male patient with a CRP concentration of 100 µg/ml (post-surgery) was used. An additional single donor serum was obtained from Innovative Research (#ICSER10ML, lot#29535-01).

Primary human neutrophils were isolated from venous donor blood collected in EDTA-containing tubes (Sarstedt, #02.1066.001) using PolymorphPrep (Progen, #1114683) with a purity of 89%–97% following manufacturer’s instructions. Venous blood for peripheral blood mononuclear cell (PBMC) isolation was collected in Lithium-Heparin collection tubes (Sarstedt, #04.1920.100) and PBMCs were isolated using Biocoll (Biochrom, #L6115) and Leucosep tubes (Greiner, #227290) following manufacturer’s instructions with minor changes. In brief, centrifugation of the blood was done at 1000 xg for 20 min, harvested PBMCs were washed with McCoy’s 5A modified medium (Thermo Fisher Scientific, #16600082) containing 2% fetal bovine serum (FBS) (Sigma, #F9665), and centrifuged at 650 xg. Final composition of isolated PBMCs was analyzed by XN-10 Hematology Analyzer (Sysmex) using the body fluid measurement program (averages: 65%–80% lymphocytes, 15%–31% monocytes, ca. 4% neutrophils).

### Crystals and Particles

Distinct crystallization protocols were used for each kind and lot of crystals. All MSU, CPPD, calcium carbonate and oxalate preparations were analyzed by FT-IR spectroscopy. The size of the crystals was microscopically determined.

MSU crystals were generated as described before (25). In brief, **MSU lot1 and 3**: 20 mM uric acid (Merck KGaA, #U0881) was dissolved by boiling in ultrapure water containing 20 mM NaOH. After cooling to 60°C, the pH was adjusted to 8 (lot1) or 9 (lot3) and the solutions were sterile filtered. Solutions were kept under mild agitation at RT until crystal formation (i.e., for 24–72 h). Crystals were harvested on a sterile filter, washed with ethanol p.a., dried overnight at 60°C, taken up in phosphate-buffered saline (PBS) at 50 mg/ml and stored at 4°C or -20°C. Lot1 was sonicated to reduce the size of the crystals (average length: lot1 = 54.8 µm, lot1 sono = 23.2 µm, lot 3 = 55 µm). **MSU lot2**: 10 mM uric acid was dissolved by boiling in ultrapure water containing 10 mM NaOH. After cooling to 60°C, the pH was adjusted to 7.7 and 500 mM NaCl was added. Crystals formed under mild agitation at RT. They were harvested and either stored as dry powder at 4°C or taken up in Hanks’ balanced salt solution (HBSS) at 50 mg/ml (average length: lot2 = 11.3 µm). **MSU lot4 and 5**: generated like MSU lot2, but at pH 7.2 (lot4) and pH 8.5 (lot5) and using 150 mM NaCl for both. Both preparations were sonicated to reduce the size of the crystals (average length: lot4 sono = 16.5 µm, lot5 sono = 9.5 µm). Polarization microscopy was used to evaluate birefringence of MSU preparations. **Triclinic-CPPD** (t-CPPD) and **monoclinic-CPPD** (m-CPPD) were prepared as previously published (26). We used sodium pyrophosphate as precursor instead of the potassium one, and calcium chloride dihydrate instead of calcium nitrate tetrahydrate (average length: m-CPPD = 10.1 µm, t-CPPD = 14.8 µm). To generate **calcium carbonate** crystals, 15 ml of ultrapure water with 50 mM CaCl_2_ and 100 mM NaOH was filled into a Petri dish (Sarstedt, #82.1473.001) and incubated at 37°C, 5% CO_2_, and 95% humidity for 24 h. Crystals were harvested, washed with ethanol p.a., and dried overnight at 37°C. They were stored at 4°C as dry powder or taken up in HBSS at 50 mg/ml (average diameter: 7.9 µm). **Calcium oxalate monohydrate** and **calcium oxalate dihydrate** were prepared as previously published (27) (average length/diameter: monohydrate = 10.6 µm, dihydrate = 4.7 µm). A second preparation of calcium oxalate was generated following the protocol of Marschner et al. (28), and crystals were harvested, washed, and stored as described above. FT-IR spectroscopy suggested this lot to be a mixture of calcium oxalate mono- and dihydrate (CaOx mix). **Cholesterol** crystals were generated as previously described by Samstad et al. (29) using 2-propanol instead of 1-propanol for dissolving cholesterol (Roth, #8866.2) (average diameter: 4.2 µm). Silicon(IV) oxide (**silica**) was purchased from Alfa Aesar (#88316.30) and the -325-mesh powder was resuspended in PBS at 20 mg/ml (average diameter: 3 µm). Aluminum Hydroxide Gel Adjuvant (**alhydrogel**) with 10 mg/ml aluminum content was purchased from Brenntag Biosector (#vac-alu-250) and used as indicated for each experiment. **Zymosan** (Sigma-Aldrich, #Z4250) was resuspended in ethanol p.a. at 30 mg/ml and stored at -20°C (average diameter: 1.7 µm). It was washed twice with HBSS before experiments. Dried ***S. cerevisiae*** (Dr. Oetker GmbH) was rehydrated in RPMI1640 for 1 h at RT, washed with and resuspended in PBS, and heat-inactivated at 80°C for 10 min before storage at -20°C (average diameter: 4 µm).

### Cell Culture

293T, HepG2, and murine M-CSF-differentiated cells were cultured in advanced Dulbecco’s modified Eagle’s medium (DMEM) (Thermo Fisher Scientific, #12491-023), and murine GM-CSF-differentiated cells in VLE RPMI 1640 (Biochrom, #FG1415), both containing 1% Penicillin/Streptomycin (Biochrom, #A2213) and 8% FBS (Sigma, #F9665). Incubation took place at 37°C, 5% CO_2_, and 95% humidity. Adherent cells were detached using Trypsin/EDTA (Sigma, #T4299). Throughout this study, HBSS containing 1.26 mM Ca^2+^, 0.9 mM Mg^2+^, and 5.5 mM D-glucose (Thermo Fisher Scientific, #14025050), as well as PBS (Sigma, #D8537) were used as indicated.

### Generation of CRISPR/Cas9 Plasmids

Three gRNAs targeting the human *LDLR* gene were designed using the Brunello library (30). Target sequences were the following: #1 = ATGAACAGGATCCACCACGA, #2 = CTTAAGGTCATTGCAGACGT, #3 = CAGAGCACTGGAATTCGTCA (please note that only #1 generated a successful knock-out). The three corresponding CRISPR/Cas9 plasmids were generated following the protocol from Ran et al. (31) using a GFP-expressing plasmid containing the Cas9 [pSpCas9(BB)-2A-GFP (PX458) was a gift from Feng Zhang (Addgene plasmid #48138; http://n2t.net/addgene:48138; RRID: Addgene_48138)].

### Transfection

HepG2 cells were seeded with 0.5 x 10^6^ cells/well into a 6-well cell culture plate. After 24 h incubation at 37°C, the cells were transfected with 1 µg CRISPR/Cas9 plasmid using PolyJet *in vitro* DNA Transfection Reagent (SignaGen, #SL100688-1) following manufacturer’s instructions. 24 h post-transfection, cells were detached using Trypsin/EDTA, washed with PBS containing 0.5% BSA (Roche, #10735086001) and 2 mM EDTA (Roth, #X986.2), and subject to single cell sorting.

### Cell Sorting and Flow Cytometry

Using the FACSAria Fusion (BD Biosciences), successfully transfected (i.e., GFP-positive) HepG2 cells were sorted. Single cells were placed in a 96-well cell culture plate containing 150 µl standard DMEM culture medium. For a minimum of 4 weeks, cells were incubated and expanded. To evaluate the success of the CRISPR/Cas9-mediated LDLR knock-out, cells were stained with a PE-labeled human LDLR antibody (Sino Biological, #10231-R301-P, 4 µg/ml) and LDLR expression was analyzed using a FACS Canto II (BD Biosciences) and BD FACSDiva software version 6.1.3 (https://www.bdbiosciences.com/en-eu). Flowing Software version 2.5.1 (Perttu Terho, University of Turku, Finland, http://flowingsoftware.btk.fi/) was used for visualization and analysis of data obtained.

### Phagocytosis of Particles Determined by Polarization Microscopy

In a 6-well cell culture plate, 0.5 x 10^6^ HepG2 cells/well were seeded. After 18 h, cells were incubated with 300 µg MSU crystals (lot2) at 37°C for 30 min. Cells were extensively washed with PBS, detached using Trypsin/EDTA, washed again, and taken up in DMEM for mounting on glass slides. The amount of phagocytes was determined by counting the cells with/without intracellular MSU crystals within 500 cells per sample using the Orthoplan polarization microscope (Leitz, #833679) with a 20-fold magnification.

### ROS Measurement

Primary human neutrophils were isolated and 2 x 10^5^ cells/well were seeded in 100 µl HBSS into a FBS-coated, white 96-well cell culture plate (Costar, #3912). HBSS stimulation mixture containing 50 µM luminol (Sigma, 123072-5G) and particles {final concentration: 0.8 µg/µl silica, 1 x 10^6^ *S. cerevisiae* particles, 3 µl alhydrogel, and 1 µg/µl of all other crystals used [cholesterol, t-CPPD, m-CPPD, calcium oxalate mono- and dihydrate, calcium carbonate, MSU (lot1), MSU (lot2)]} was prepared. Then, 50 µl stimulus mixture were added to the cells and ROS production was immediately detected for 60 min using the Berthold Orion L Microplate Luminometer and Simplicity software 4.20 (https://www.berthold.com/en/).

### Generation of Recombinant Fc-Fusion Proteins

The generation of Fc-fusion proteins for mouse Clec7A, mouse Clec12A, and human Clec12A has been previously described (24). For murine MARCO (NCBI: NP_034896.1, aa: 83-518) and human MARCO (NCBI: NP_006761.1, aa: 79-520), the cDNA of the extracellular domains (Origene, #MR222739 and #RC205625, respectively) were amplified by PCR and verified by restriction digest. Primers used in 5’-3’ direction: mMARCO forward = ATGTGCTGTGGCAATGGATC and reverse = GGAGCATTCCACACCCG; hMARCO forward = ATGTATTTCCTCAATGACACTCTG and reverse = GACGCTGCACTCCACG. The cDNAs were fused to the C-terminus of human IgG1-Fc in the expression vector pFUSE-hIgG1-Fc2 (InvivoGen, #pfuse-hg1fc2). After sequence verification, constructs were transfected into 293T cells (DSMZ, #ACC 635) using PolyJet Transfection Reagent as described above. Supernatants were harvested 48 and 72 h post transfection, pooled, sterile filtered, and stored at 4°C until usage as a source of recombinant proteins. Correct protein secretion was verified by Western blot analysis.

### Murine Cells and Differentiation

LDLR-deficient mice (B6.129S7-*Ldlr^tm1Her^*/J; maintained on a C57BL/6 background) and C57BL/6 control animals were originally obtained from The Jackson Laboratory (https://www.jax.org/strain/002207) and kindly donated by Prof. Sibylle von Vietinghoff. Venous blood was drawn from animals through the eye vein. Following 30 min incubation at RT, serum was collected by centrifugation at 10000 xg for 2 min. Sera of a minimum of three animals were pooled and frozen at -80°C. Total bone marrow cells were isolated from femur and tibia of LDLR KO and WT mice using standard procedures. For M-CSF-differentiated cells, 7.5 x 10^6^ bone marrow cells were incubated for 7 days in 10 ml DMEM containing 25 ng/ml M-CSF (Peprotech, #315-02). On day 3, 10 ml fresh medium were added. For GM-CSF-differentiated cells, 5 x 10^6^ bone marrow cells were incubated for 9 days in 10 ml VLE RPMI 1640 (Biochrom, #FG1415) including 10 ng/ml GM-CSF (Peprotech, #315-03), with 8 ml fresh medium being added on day 3 and 6. M-CSF- and GM-CSF-differentiated cells were harvested by incubation with ice-cold PBS for 15 min at 4°C and rinsing the plates firmly.

To collect bone marrow cells and blood, animals were euthanized according to the guidelines of the German animal protection act §4 (Tötung zu wissenschaftlichen Zwecken/sacrifice for scientific purposes). All procedures were carried out in accordance with the relevant guidelines and regulations from the Lower Saxony State Office for Consumer Protection and Food Safety and Hannover Medical School, Germany.

### Phagocytosis of Particles Determined Using Flow Cytometry

M-CSF- and GM-CSF-differentiated cells were harvested as described above. 3 x 10^5^ cells/well were seeded in 300 µl medium in a 48-well cell culture plate (Corning, #3548) and incubated for 18 h. Then, 150 µg crystals or 1 x 10^6^ *S. cerevisiae* particles were added to the cells and mixed firmly. Following incubation at 37°C for 30 min, cells were harvested using Accutase x1 (Biolegend, #423201) and phagocytosis was stopped by placing the cells on ice. Cells were stained with Mouse Fc-Block (BioLegend, #101320, 5 µg/ml) and AlexaFluor647 CD11b-antibody (Biolegend, #101218, 2.5 µg/ml) in PBS containing 0.5% BSA and 2 mM EDTA. The uptake of particles was evaluated by assessing changes in granularity (SSC = sideward scatter) of the CD11b-positive cells using flow cytometry (22, 32, 33).

### Inflammasome Activation

M-CSF- and GM-CSF-differentiated cells were harvested as described above. 1.5 x 10^5^ cells/well were seeded in 100 µl medium in a 96-well cell culture plate (Corning, #3799) and incubated for 18 h. Following pre-incubation for 3 h with 20 ng/ml LPS (Alexis, #581-012-L002), cells were stimulated with crystals/particles for 4 h [final concentration: 6.9 µM nigericin (Invivogen, #Tlrl-nic), 0.8 µg/µl silica, 1 x 10^6^ *S. cerevisiae* particles, and 1 µg/µl of all other crystals [cholesterol, t-CPPD, MSU (lot1), MSU (lot2)]. Supernatants were harvested and IL-1β secretion was determined by ELISA analysis.

### Induction of IL-1β Production

PBMCs were isolated as described above. 2 x 10^5^ cells/well were seeded in 100 µl McCoy’s 5A modified serum-free medium containing 1% penicillin/streptomycin in a 96-well cell culture plate (Corning, #3799). HBSS stimulation mixture containing particles {final concentration: 6.9 µM nigericin, 0.8 µg/µl silica, 1 x 10^6^ *S. cerevisiae* particles, 3 µl alhydrogel, 1 µg/µl of all other crystals used [cholesterol, t-CPPD, m-CPPD, calcium oxalate mono- and dihydrate, calcium carbonate, MSU (lot1), MSU (lot2)]} was prepared. Then, 50 µl stimulus mixture were added to the cells, supernatants were collected after 16 h incubation, and IL-1β production was quantified using ELISA.

### Quantification of IL-1β

Human and murine IL-1β was quantified using uncoated ELISA Kits (Thermo Fisher Scientific, #88-7261-88 and #88-7013-88, respectively) following manufacturer’s instructions.

### Purification of Particle-Binding Proteins

Unless otherwise stated, 50 µl of human serum were incubated with 2 mg crystals, 1 mg zymosan, or 5 x 10^6^ *S. cerevisiae* particles at 37°C for 30 min with agitation (1300 rpm). After incubation, supernatant and particles were separated by centrifugation at 4000 xg for 2 min. While supernatant was diluted 1:20 in 1x SDS-PAGE sample buffer (+DTT), particles were extensively washed with HBSS (saturated with uric acid) and bound proteins were eluted by adding 100 µl of 4x SDS-PAGE sample buffer (+DTT). All samples were heated at 70°C for 10 min and subject to SDS-PAGE and Western blot analysis.

### Recombinant Protein Binding Analyzed Using Flow Cytometry

For flow cytometric analysis, CPPD preparations, calcium oxalate mono-, and dihydrate, silica, and calcium carbonate were filtered through a SmartStrainers (30 µm) by Miltenyi Biotec (#130-098-458) to remove too large crystals.

**Fc-fusion proteins** in 50 µl DMEM were incubated with 150 µg MSU crystals at RT for 60 min. Crystals were washed with HBSS and bound Fc-fusion proteins were stained with AlexaFluor488 goat anti-human IgG (Jackson ImmunoResearch Europe Ltd., #109-545-008, 3.75 µg/ml) at RT for 30 min. **His-tagged recombinant proteins** (5 µg/ml in HBSS) were incubated with 150 µg crystals or 1 x 10^6^ *S. cerevisiae* particles at 37°C for 30 min. We used recombinant human MARCO (R&D Systems, #7586-MA-050), human LDLR (Sino Biological, #10231-H08H-50), human CD32b (Sino Biological, #10259-H08H), and murine LDLR (Sino Biological, #50305-M08H). After washing with HBSS, bound proteins were detected at 4°C for 30 min using PE mouse anti-His Tag (Biolegend, #362603, 0.24 µg/ml).

Purified human CRP (Merck KGaA, #AG723) bound to crystals was detected by incubation with a rabbit CRP antibody (Merck KGaA, #235752, 10 μg/ml) at 4°C for 1 h, and subsequent incubation with PE donkey anti-rabbit IgG (BioLegend, #406421, 2 μg/ml) for 30 min at 4°C.

For both sets of tagged recombinant proteins and purified CRP, particles were washed with and taken up in 5% BSA in HBSS before using FACS Canto II (BD Biosciences) to measure the fluorescence of the particles. BD FACSDiva software version 6.1.3 (https://www.bdbiosciences.com/en-eu) was used for data acquisition and calculation of mean or median fluorescence intensity as indicated in the figure legends.

### Fluorescence Microscopy

Around 100–200 µg of MSU, CPPD, silica, cholesterol, and calcium carbonate crystals, or 1 x 10^6^ *S. cerevisiae* particles were incubated either in human serum or HBSS at 37°C for 30 min. After washing with 5% BSA in HBSS, the particles were incubated with recombinant proteins as described above for flow cytometry. Fc-fusion proteins were stained the same way. Recombinant His-tagged proteins were detected using purified anti-His Tag (Biolegend, #362602, 5 µg/ml) and Alexa Fluor488 goat anti-mouse IgG (Biolegend, #405319, 5 µg/ml) binding at 4°C for 30 min each. Following a last washing step, the particles were mounted in Immunoselect Antifading Mounting Medium (Dianova, #SCR-038447).

Images were either acquired using an Olympus IX81 inverted microscope with a UPlanSApo 60x/1.35 Oil objective and Cell^R^ software (version 3.2; https://www.olympus-lifescience.com/en/software/) or a Zeiss 980 Airyscan 2 with an alpha Plan-Apochromat 63x/1.46 Oil Korr M27 objective in combination with Zeiss ZEN System software (blue edition version 3.0; www.zeiss.com/microscopy/int/products/microscope-software/zen.html). Brightness was adjusted, pseudo-color was inserted in the grayscale image, and scale bar was added using ImageJ (version 1.52d) (https://imagej.nih.gov/ij/) (25).

### LDL Depletion in Serum

Human serum containing 10 µg/ml purified human CRP (Merck KGaA, #AG723) was vigorously mixed with HBSS or LipoSep Immunoprecipitation Reagent (Bio-Connect Diagnostics, #LS-01) at a 1:1 ratio. Following incubation at RT for 10 min the mixture was centrifuged at 12000 xg for 10 min. The supernatant (= LDL-depleted serum) was harvested and used for purification of particle-binding proteins or to opsonize crystals at 37°C for 30 min with agitation (1300 rpm). Protein binding was subsequently analyzed using flow cytometry.

### SDS-PAGE and LC-MS

SDS-PAGE was performed with 4%–20% precast gels in Tris-based buffer system from SERVA Electrophoresis (#43289.01) according to the manufacturer’s instructions. PageBlue Protein Staining Solution (Fermentas, #R0571) was used for coomassie-staining the gels, images were taken with iPhone 6s (Apple) and the contrast was adjusted using ImageJ (version 1.52d). Global identification and quantification of MSU- and zymosan-binding proteins in the presence of different donor sera was done as previously described (25). In brief, eluted proteins were reduced with DTT, alkylated with acrylamide, and separated using SDS-PAGE (4%–20%, Sigma-Aldrich). Whole lanes were cut into three individual slices and proteins therein were in-gel digested with trypsin. Generated peptides were analyzed using an LC-MS system consisting of an Orbitrap Velos mass spectrometer coupled to an Ultimate 3000 RSLC nanoflow system (Thermo Fisher Scientific). Raw data were analyzed with the Andromeda search engine implemented in MaxQuant software (version 1.5.3.30; www.maxquant.org). Proteins were identified based on a false discovery rate (FDR) of less than 0.01 on protein and peptide level.

### Western Blot Analysis

As published before (25), proteins were separated by SDS-PAGE and transferred to nitrocellulose membranes (GE Healthcare, #10600003). Membranes were blocked in TBST + 5% BSA, incubated in primary antibody in TBST + 5% BSA o/n at 4°C. After incubation with HRP-coupled secondary antibodies (Cell Signaling Technology, #7074, #7076S), blots were subjected to ECL reaction. Images were acquired using ChemoStar (INTAS Science Imaging Instruments GmbH) and contrast was adjusted using ImageJ (version 1.52d). The band with the strongest signal was set to maximum (black). Primary antibodies and dilution were: ApoB (#sc-393636; 1:200) from Santa Cruz Biotechnology, ApoE (Proteintech, #66830-1-Ig, 1:20000), ApoAI (Proteintech, #14427-1-AP, 1#2000), and CRP (Proteintech, #66250-1-Ig; 1:10000).

### Statistical Analysis

Unless otherwise stated in the figure legends, a paired two-sided t-test was performed for statistical comparison of two groups. All analyses were performed using GrapPad Prism version 5.02 and 8.4.3 (GraphPad Software; www.graphpad.com/scientific-software/prism/). A p value of < 0.05 was considered statistically significant. *, p < 0.05; **, p < 0.01; ***, p < 0.001; ns = not significant.

## Results

### LDLR and MARCO Bind to MSU Crystals

In a previous study, we identified CRP as a major MSU crystal binding protein in human body fluids by mass spectrometry (25). Here, we again used mass spectrometry to also identify low abundant MSU crystal binding proteins in human serum. Since the inflammatory response induced by MSU crystals and fungi is very similar, zymosan (well characterized cell wall preparation of *S. cerevisiae*) was used as a control. Normal human serum and serum from an individual with an acute phase reaction (systemic inflammation) were incubated with MSU crystals or zymosan. After washing away the unbound proteins, bound proteins were eluted with denaturating SDS buffer and separated *via* SDS-PAGE. Similar to our previous data using synovial fluid, visualization of the proteins by coomassie staining showed a mostly distinct pattern between MSU-bound proteins and zymosan-bound proteins (**Figure S1A**). The whole lanes were analyzed by liquid chromatography-mass spectrometry (LC-MS) and label-free relative quantification of the binding proteins was done using MaxQuant. This experiment confirmed our previous finding that CRP binds to MSU but not to zymosan (25).

**Figure 1:**
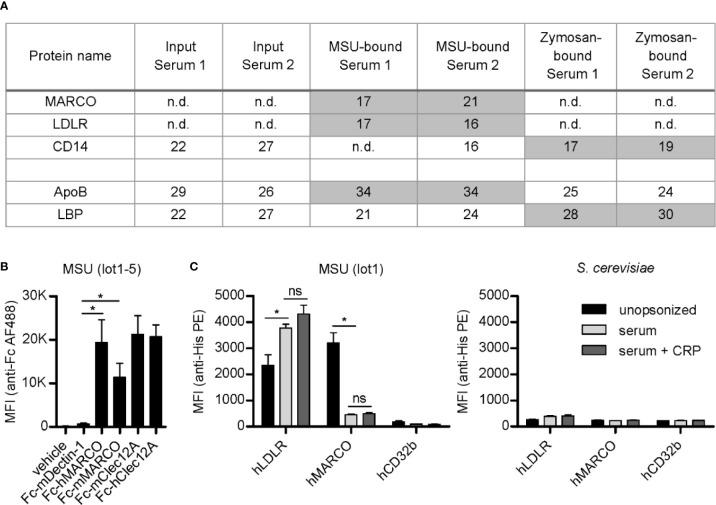
LDLR and MARCO bind to MSU crystals. **(A)** Normal human serum (serum 1) and serum from an individual with an acute phase reaction (serum 2) were incubated with MSU crystals (lot1) or zymosan at 37°C for 45 min. Bound proteins were eluted, separated by SDS-PAGE, and subjected to LC-MS analysis. The relative intensity (log2-intensity) of transmembrane receptors and their respective ligands (ApoB is a ligand of LDLR and LBP is a ligand of CD14) bound to MSU crystals or zymosan is shown, compared to the intensity in the input serum; n.d., not detected. **(B)** Five distinct MSU crystal preparations (lot1-5) were incubated with recombinant Fc-proteins at room temperature (RT) for 60 min (vehicle = DMEM, Fc-mDectin-1, Fc-hMARCO, Fc-mMARCO, Fc-mClec12A, Fc-hClec12A). Crystals were washed with HBSS (always containing Ca^2+^); bound Fc-fusion proteins were stained with anti-human IgG AlexaFluor488 and the fluorescence of the particles was analyzed using a flow cytometer. An unpaired, two-sided t-test was used for statistical evaluation (ns = not significant, *p < 0.05); mean fluorescent intensity (MFI) and standard error of the mean (SEM) are shown. **(C)** MSU crystals (lot1; left) or *S. cerevisiae* particles (right) were incubated in HBSS (unopsonized) or human serum from three individual healthy donors with or without the addition of 40 µg/ml CRP at 37°C for 30 min. After washing with HBSS the particles were incubated with 5 µg/ml recombinant His-tagged protein in HBSS + 5% BSA at 4°C for 60 min (hLDLR, hMARCO, hCD32b). Bound proteins were stained with anti-His Tag PE and the fluorescence of the particles was analyzed using a flow cytometer. An unpaired, two-sided t-test was used for statistical evaluation (ns = not significant, *p < 0.05); MFI and SEM are shown.

We aimed to identify soluble serum proteins involved in the opsonization of MSU crystals. Unexpectedly, we found transmembrane receptors (MARCO, LDLR, and CD14) bound to MSU crystals or zymosan (**Figure 1A**). MARCO and LDLR were neither detected in the input sera nor bound to zymosan but were detected in both purifications of MSU binding proteins, indicating a strong enrichment by MSU crystals and specific binding. The intensity of LDLR and CD14 correlated with their corresponding ligands apolipoprotein B (ApoB) and LPS-binding protein (LBP), respectively (**Figure 1A**). These data suggest strong binding of MARCO and LDLR to MSU crystals, with LDLR potentially binding to ApoB coated on the crystals. CD14 was hardly detectable on MSU crystals and bound more strongly to zymosan, potentially *via* coated LBP. Therefore, we focused on the interaction of MARCO and LDLR with crystalline structures and their potential roles in cellular responses to crystals.

To test binding of MARCO to crystals, we generated both human and murine MARCO-Fc-fusion proteins (Fc-hMARCO and Fc-mMARCO, respectively). Both bound strongly to five distinct preparations of unopsonized MSU crystals (lot1-5) (**Figure 1B**). The binding was generally at least as strong as the known MSU receptor Clec12A, indicating MARCO may be a conserved receptor for these crystals. The well-known receptor for fungal beta-glucans Dectin-1 (also known as Clec7A) served as negative control.

Using commercial recombinant His-tagged versions of human LDLR and MARCO (hLDLR and hMARCO, respectively), we found that LDLR indeed bound to opsonized crystals. It did, however, also bind to unopsonized MSU crystals (**Figure 1C**, left panel). Moreover, we could confirm the binding of MARCO to unopsonized MSU crystals, which was strongly reduced by pre-incubation of the crystals with human serum, indicating that MARCO might be a receptor for unopsonized particles. As a negative control we used a His-tagged version of the low affinity IgG receptor hCD32b which has not yet been associated with crystal binding. Moreover, none of the tested receptors bound to *S. cerevisiae* (**Figure 1C**, right panel). Even though CRP strongly binds to and recruits other proteins to the surface of MSU crystals (25), addition of purified CRP to the sera used for opsonization did not have a significant effect on binding of MARCO or LDLR to the crystals (**Figure 1C**, left panel). This indicates that CRP is neither recognized by these receptors nor does it significantly enhance the blocking of the crystal surface by serum components.

### MARCO Binds to Unopsonized Crystals

**Figure 2:**
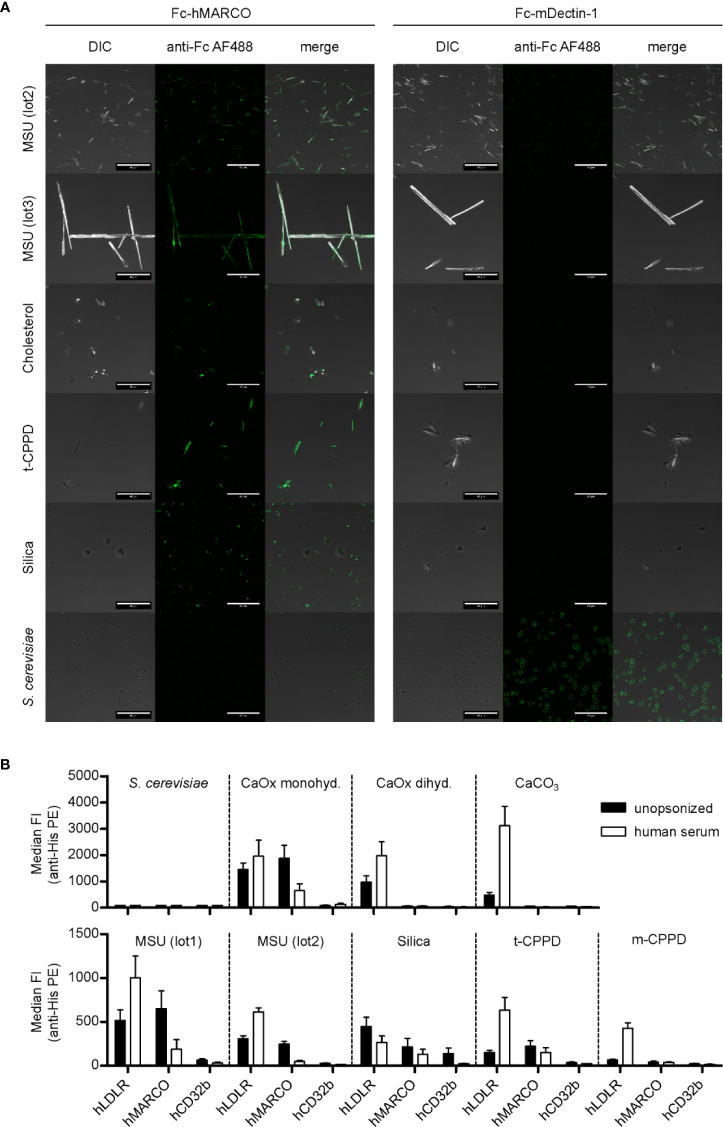
MARCO binds to unopsonized crystals. **(A)** Confocal microscopy of MSU (lot2, lot3), cholesterol, t-CPPD, silica, and *S. cerevisiae.* Particles were incubated at RT for 60 min with either Fc-hMARCO or Fc-mDectin-1 recombinant protein. After washing with HBSS, bound proteins were visualized using anti-human IgG AlexaFluor488 (anti-Fc AF488, green). Representative of at least two independent experiments; DIC = digital interference contrast; scale bar = 40 µm. **(B)** Indicated crystals were incubated in HBSS (unopsonized) or human serum from three individual healthy donors at 37°C for 30 min. After washing with HBSS the particles were incubated with 5 µg/ml recombinant His-tagged protein in HBSS + 5% BSA at 4°C for 60 min (hLDLR, hMARCO, hCD32b). Bound proteins were stained with anti-His Tag PE and the fluorescence of the particles was analyzed using a flow cytometer. Median fluorescent intensity (FI) and SEM are shown. Negative control = *S. cerevisiae*.

In **Figure 1**, we showed that MARCO and LDLR bind to MSU crystals. To asses if this interaction is specific, we analyzed their binding to a broader spectrum of crystals known for causing crystallopathies. First, we used confocal microscopy to visualize the binding of Fc-hMARCO to unopsonized particles and saw a strong fixation on the surface of two distinct preparations of MSU, triclinic CPPD (t-CPPD), silica crystals, and to a lesser extent to cholesterol crystals (**Figure 2A**, left panel). Again, Dectin-1 served as a control and bound strongly to heat inactivated yeast particles (*S. cerevisiae*) but not to crystalline structures (**Figure 2A**, right panel).

To quantify the binding of the receptors to the crystals, we analyzed binding of His-tagged proteins (hMARCO, hLDLR, and hCD32b) to unopsonized and opsonized particles using a flow cytometer (**Figure 2B**). None of the receptors was binding to *S. cerevisiae* (**Figure 2B**) and zymosan (data not shown). In addition to the preparations shown in **Figure 2A**, we observed binding of hMARCO to calcium oxalate monohydrate. Strikingly, in all these samples it bound strongly to the unopsonized crystals, but much weaker to the ones incubated in human sera. This indicates that hMARCO binds directly to the naked surface of the crystals and that serum proteins or other constituents bound to the crystals block the hMARCO binding site. Interestingly, incubation with hLDLR led to an opposite effect for most crystals. hLDLR bound to all crystalline structures tested, but in the majority of cases much stronger to the opsonized versions compared to the unopsonized ones (**Figure 2B**). Therefore, it is reasonable to assume that the binding of LDLR to crystals is mainly indirect. On a side note, hLDLR binds to silica in two distinct populations, which indicates that the silica particles used are not a homogenous entity (**Figure S1B**).

Together, this shows that the extracellular domain of human MARCO directly binds to several crystals that are associated with disease (MSU, calcium oxalate monohydrate, silica, and t-CPPD). Furthermore, it raises the question which serum proteins might mediate the indirect binding of hLDLR to opsonized crystals [calcium oxalate dihydrate, calcium carbonate (CaCO_3_), MSU, t- and monoclinic CPPD (m-CPPD)].

### LDLR Binds Both Naked Crystals and LDL-Coated on Crystals

**Figure 3:**
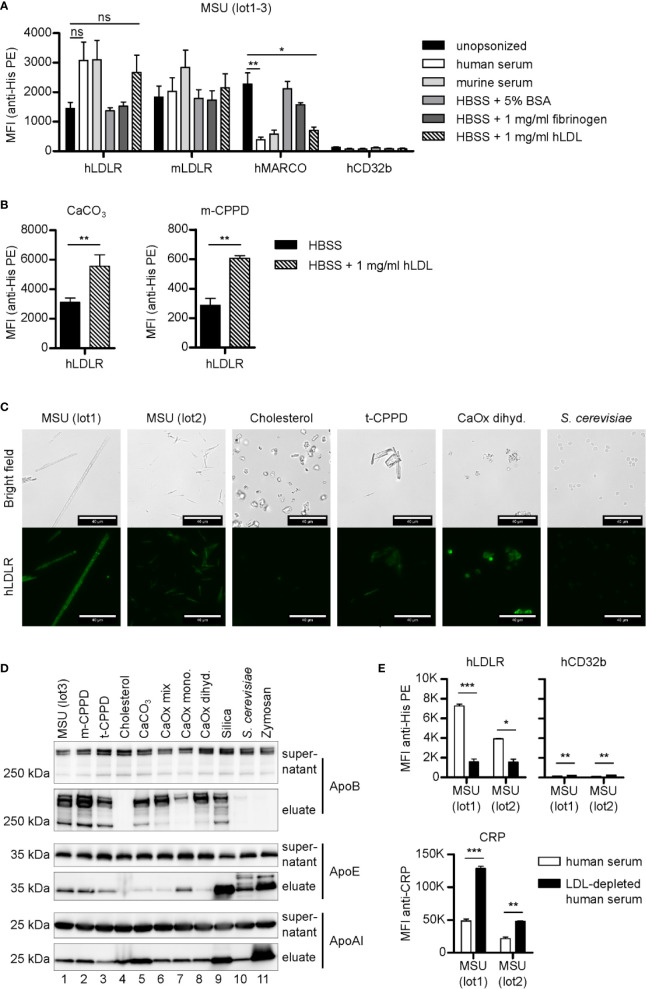
LDLR binds to both opsonized and unopsonized crystals. **(A)** MSU crystals (lot1-3) were opsonized in different solutions (unopsonized (HBSS), human serum, murine serum, or HBSS containing 5% BSA, 1 mg/ml fibrinogen, or 1 mg/ml LDL) at 37°C for 30 min. After washing with HBSS, the particles were incubated with 5 µg/ml recombinant His-tagged protein in HBSS + 5% BSA at 4°C for 60 min (hLDLR, mLDLR, hMARCO, hCD32b). Bound proteins were stained with anti-His Tag PE and analyzed using a flow cytometer. An unpaired, two-sided t-test was used for statistical evaluation (ns = not significant, *p < 0.05, **p < 0.01 ); MFI and SEM are shown. **(B)** Calcium carbonate and m-CPPD crystals were pre-incubated in HBSS or HBSS-containing 1 mg/ml hLDL and then incubated with hLDLR and stained as described in **(A)**. An unpaired, two-sided t-test was used for statistical evaluation (ns = not significant, **p < 0.01). Mean MFI and SEM of n=3 samples is shown. **(C)** Indicated crystals were opsonized in human serum at 37°C for 30 min. Incubation with recombinant LDLR was done as in **(A)**. Protein binding was analyzed using mouse anti-His Tag plus goat anti-mouse AlexaFluor488. Fluorescence of the samples was detected by fluorescent microscopy; scale bar = 40 µm. Representative of three independent experiments. **(D)** Indicated crystals were opsonized with human serum at 37°C for 30 min. After incubation, particles and supernatant were separated by centrifugation: the supernatant was collected, while the particles were extensively washed with HBSS to remove unbound proteins. Bound proteins were eluted. Both supernatants and eluates were subjected to Western blot analysis using ApoB, ApoAI, and ApoE antibodies. **(E)** Two distinct preparations of MSU crystals (lot1, lot2) were incubated with human serum or LDL-depleted human serum (both containing 10 µg/ml CRP) at 37°C for 30 min. Incubation with recombinant proteins (hLDLR and hCD32b) as well as detection and analysis of the bound proteins was performed as described in **(A)**; MFI and SEM are shown. CRP binding was analyzed using CRP antibody and anti-rabbit-AlexaFluor488. Fluorescence of the crystals was detected by flow cytometry (MFI anti-CRP +SEM). A paired, two-sided t-test was used for statistical evaluation. (*p < 0.05, **p < 0.01, ***p < 0.001).

MARCOs ability to bind MSU crystals appeared to be conserved between species (**Figure 1B**). To test if the binding of LDLR is also conserved between species, we next compared binding of recombinant His-tagged human and mouse LDLR to MSU crystals. Both bound to naked crystals as well as crystals opsonized with either human or mouse serum (**Figure 3A**).

Since it is likely that LDLR binds to its cognate ligand ApoB or ApoE on the surface of the opsonized crystals, we opsonized the crystals in buffer containing 5% bovine serum albumin (BSA), 1 mg/ml fibrinogen, or 1 mg/ml human LDL. Both LDLRs bound to MSU crystals opsonized with these solutions, however, only LDL was able to inhibit binding of MARCO (**Figure 3A**), indicating only LDL-binding is strong enough to block the binding sites on the crystal surface (**Figure 3A**). Together, this shows that LDLR binds to both naked and LDL-coated MSU crystals. When we coated calcium carbonate and m-CPPD crystals with LDL and tested binding of LDLR, we found enhanced binding to LDL-coated crystals (**Figure 3B**), showing that depending on the crystal type, LDL-coating can strongly enhance LDLR binding.

We visualized the binding of hLDLR to a broad spectrum of serum opsonized crystals by fluorescent microscopy and could verify binding to all opsonized particles except cholesterol (**Figure 3C**). Similar binding of LDLR was observed when the crystals were opsonized with LDL, again showing hardly any binding to cholesterol crystals (**Figure S2**). We then purified serum proteins that bound to the various crystals and subjected them to SDS-PAGE and Western blotting. In line with the notion that LDL mediates binding of LDLR to crystals, we saw strong binding of ApoB, the apolipoprotein of LDL particles, to all crystals but cholesterol (**Figure 3D**). ApoE, which has been shown to bind to MSU crystals (21) and is also a ligand of LDLR (34), bound to all crystals with varying degree but most strongly to the fungal particles and silica. The apolipoprotein of HDL, ApoA1, also bound to all crystals with varying intensity, but most strongly to zymosan as we have already seen in our previous study (25).

To test if LDL is indeed required for LDLR binding to opsonized crystals, we depleted LDL in three different human sera by immunoprecipitation. We confirmed depletion of ApoB but not ApoE or ApoA1 by Western blot analysis (**Figure S3**) and used the serum for opsonization of MSU crystals. Binding of hLDLR to MSU crystals opsonized with LDL-depleted serum was strongly reduced compared to MSU crystal opsonized with LDL-containing serum (**Figure 3E**). Binding of the known MSU recognition molecule CRP was significantly stronger on MSU crystals in the absence of LDL, suggesting competition with LDL for binding sites. On a very low level even the negative control receptor CD32b bound significantly stronger in the absence of LDL, suggesting LDL is the main serum component blocking the crystal surface.

Together, these data suggest that LDLR is a conserved receptor for recognition of opsonized crystalline structures and that it mainly binds to LDL on opsonized MSU crystals.

### LDLR Has No Influence on Inflammasome Activation and Phagocytosis After Stimulation With Crystals in M-CSF- or GM-CSF-Differentiated Murine Bone Marrow Cells

We showed in **Figures 3A** that murine LDLR binds to MSU crystals opsonized with murine serum. To elucidate the role of LDLR in cellular responses to crystals, we studied phagocytosis of the crystals and the production of the key cytokine IL-1β utilizing murine bone marrow cells lacking LDLR.

**Figure 4:**
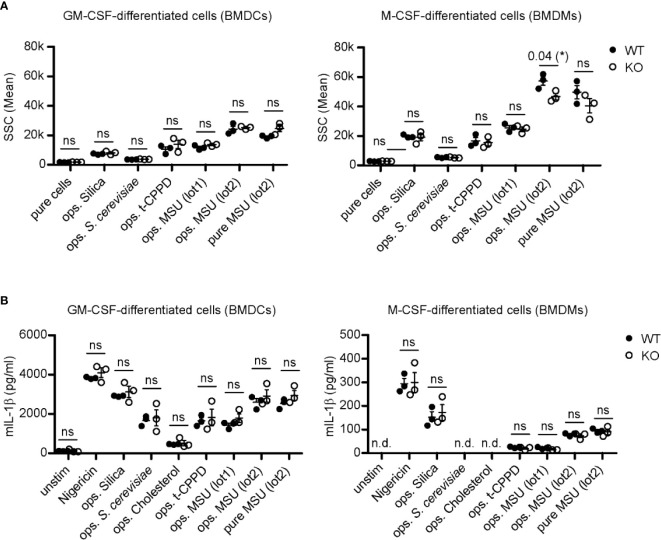
LDLR has no influence on inflammasome activation and phagocytosis after stimulation with crystals in M-CSF- or GM-CSF-differentiated murine bone marrow cells. **(A)** Primary murine GM-CSF- and M-CSF-differentiated bone marrow cells [bone marrow-derived dendritic cells (BMDCs) and macrophages (BMDMs), respectively] were incubated with 150 µg crystals [silica, t-CPPD, MSU (lot1), MSU (lot2)] or 1 x 10^6^ *S. cerevisiae* particles for 30 min at 37°C. Particles were incubated in murine pool serum at 37°C for 30 min (ops.) and washed with HBSS before adding to the cells; unopsonized crystals (pure) were stored in HBSS. Uptake of particles was evaluated by assessing the change in granularity (SSC = sideward scatter) of CD11b-positive cells using flow cytometry. An unpaired, two-sided t-test was used for statistical evaluation (ns = not significant, *p < 0.05, 3 WT vs. 3 LDLR KO mice). **(B)** Primary murine GM-CSF- and M-CSF-differentiated cells (BMDCs and BMDMs, respectively) were pre-incubated with 20 ng/ml LPS for 3 h before adding 0.8–1 mg/ml crystals/particles [nigericin, silica, *S. cerevisiae*, cholesterol, t-CPPD, MSU (lot1), MSU (lot2)]. Opsonization of particles was performed as described in **(A)** at 37°C for 30 min. After 4 h, the supernatant of the cells was collected and IL-1β secretion was determined by ELISA analysis. An unpaired, two-sided t-test was used for statistical evaluation (n.d., not detectable, ns, not significant, 3 WT vs. 3 LDLR KO mice).

Granulocyte-macrophage colony-stimulating factor (GM-CSF)-differentiated murine bone marrow cells (classically called BMDCs) or macrophage colony-stimulating factor (M- CSF)-differentiated murine bone marrow cells (BMDMs) lacking LDLR (KO) and wild type (WT) control cells were incubated with distinct crystals/particles. Phagocytosis was evaluated by assessing the increase in cellular granularity (sideward-scatter) using flow cytometry only in CD11b-positive cells to distinguish cells from crystals (**Figure S6**). The percentage of CD11b-positive cells in the differentiated bone marrow cells of WT and LDLR KO cells was similar (**Figure S1C**). In GM-CSF-differentiated cells lack of LDLR KO had no effect on the uptake of particles (**Figure 4A**, left panel). M-CSF-differentiated cells showed similar results, with the exception of a slightly (though significantly) reduced ability of phagocytosis of small opsonized MSU crystals (lot2) (**Figure 4A**, right panel). This suggests that LDLR is hardly involved in phagocytosis of opsonized particles by murine immune cells, while it is potentially able to endocytose very small MSU crystals.

In order to investigate phagocytosis in human—albeit not primary—cells, we targeted the LDLR gene by CRISPR/Cas9 in the HepG2 cell line. Only one out of three gRNAs was successful and generated a single KO clone (**Figure S1D**). Although these cells were lacking LDLR, phagocytosis of pure and opsonized MSU crystals was largely unaltered in these cells. While opsonization of the particles collectively reduced the uptake, absence of LDLR had no effect on phagocytosis (**Figure S1E**), suggesting that LDLR at least does not play a major role in the endocytosis of MSU crystals in these cells.

To analyze inflammasome activation, GM-CSF- or M-CSF-differentiated bone marrow cells from LDLR KO and WT mice were primed with LPS, incubated with crystalline structures, and production of IL-1β was measured. Depending on the type of crystal, we observed activation of the inflammasome to different degrees, e.g., incubation with MSU crystals or silica generally led to more IL-1β production than cholesterol. However, this activation seemed to be opsonization-independent and more importantly, also independent of LDLR (**Figure 4B**).

Overall, these data indicate that LDLR does not play an obvious role in immune cell activation by crystals, although the data from one type of crystals suggest that it may principally be capable of enhancing crystal phagocytosis.

### LDL Opsonization Regulates Crystal-Induced ROS and IL-1β Production

As shown before, activation of neutrophils by MSU crystals is strongly reduced if the crystals are opsonized with LDL or ApoB (20). However, it is unclear if LDL also regulates neutrophil activation by other crystals and if it regulates IL-1β production. Therefore, we evaluated the role of LDL opsonization of MSU and other crystals in the production of reactive oxygen species (ROS) and IL-1β in human neutrophils and peripheral blood mononuclear cells (PBMCs), respectively.

**Figure 5:**
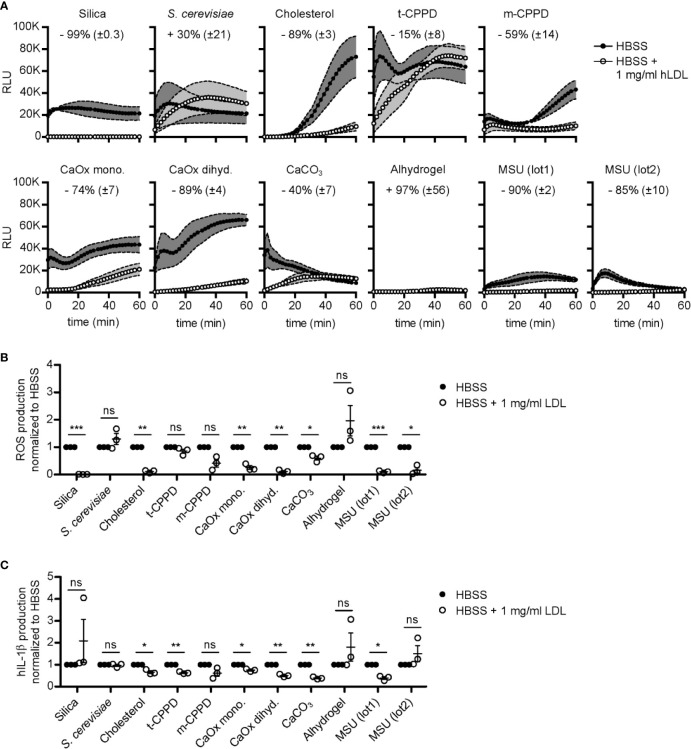
LDL opsonization regulates crystal-induced ROS and IL-1β production. **(A)** Crystals as indicated were incubated in HBSS (unopsonized) or HBSS containing 1 mg/ml human LDL at 37°C for 30 min. A stimulation mixture of these particles and luminol was then added to human neutrophils and ROS production was immediately detected for 60 min. Thereby, the emitted light (RLU = relative light units) is an indirect measure of ROS produced. Mean of kinetics of ROS production curves of three independent donors are shown with ± SEM illustrated as grey envelopes around the curves. The mean percentage change ( ± SEM) in ROS production after stimulation with LDL-opsonized particles is depicted as numbers in each graph; data combined from three independent donors. **(B)** ROS production was normalized to HBSS for each crystal separately and a paired, two-sided t-test was used to compare HBSS vs. HBSS + 1 mg/ml human LDL (ns = not significant, *p < 0.05, **p < 0.01, ***p < 0.001; three independent donors). **(C)** IL-1β production was normalized to HBSS for each crystal and a paired, two-sided t-test was used to compare both groups as in **(B)**. ns = not significant, *p < 0.05, **p < 0.01, ***p < 0.001; three independent donors.

We isolated primary human neutrophils and incubated them with a broad spectrum of crystals coated with or without LDL as indicated in **Figure 5A**. When looking at the single values of relative light units (RLU), we saw that the crystals induce different ROS kinetics (**Figure 5A**). Interestingly, the two distinct MSU preparations (lot1 and lot2) reached a similar peak of ROS production but do so at different time points. ROS induced by MSU (lot2) peaks—but also declines—faster, which is probably due to the smaller size of the MSU crystals in this preparation. We also confirmed the ROS-reducing properties of LDL-coated MSU crystals (by 85%–90%). Intriguingly, LDL opsonization had a similarly strong reducing effect on ROS production induced by silica, cholesterol, and calcium oxalate dihydrate crystals (**Figures 5A, B** **)**. For calcium oxalate monohydrate, m-CPPD, and calcium carbonate, the reduction was around 74%–40%. Only for *S. cerevisiae* and alhydrogel LDL incubation did not significantly alter ROS production (**Figure 5B**). Together these data show that LDL-coating specifically inhibits neutrophil activation by a diverse range of crystals, but not fungi.

To test, if LDL also inhibits production of IL-1β, we isolated PBMCs and stimulated them with the same broad spectrum of crystals (unopsonized and LDL-coated, respectively) as in **Figure 5A**. PBMCs produced IL-1β to various levels depending on different crystal types and sizes, as depicted for the cells of three distinct donors (**Figure S4**). *S. cerevisiae* incubation led to the highest IL-1β production, independent of LDL (**Figure S4**). For most crystals tested, opsonization with LDL reduced the production of IL-1β (cholesterol, t-CPPD, calcium oxalate mono- and dihydrate, calcium carbonate, and MSU (lot1)) (**Figure 5C**). Interestingly, IL-1β production was not inhibited by LDL coated on MSU (lot2) or silica, although LDL very effectively blocked ROS production induced by the same crystals (**Figures 5A, B** **)**. It is important to note that even sonicated MSU (lot1) (mean length before sonication: 54 µm; after sonication: 23 µm) crystals are much larger than MSU (lot2) (mean length 11.3 µm).

In summary, these data show that coating of various crystals with LDL strongly inhibits ROS production by neutrophils, while the impact of LDL on IL-1β production by non-primed PBMCs depends on structural aspects of the crystals, most likely their size.

## Discussion

In an extension of previous studies we compared the serum proteins bound to MSU crystals to those bound to the microbial pattern zymosan. We confirmed binding of LDL (19) and CRP to MSU crystals, but not to zymosan (25). Unexpectedly, within the bound serum proteins we identified the transmembrane receptors MARCO, LDLR, and CD14 as proteins binding to MSU or zymosan. CD14 is known to exist in a membrane-bound and a soluble form (35) and we also found it in the input serum. The fact that we newly identified MARCO and LDLR on MSU crystals might be due to the ever increasing sensitivity of LC-MS methodology and the potential possibilities for their release, i.e., cleavage from the cell surface or secretion in a soluble form.

For MARCO, a class A scavenger receptor (SR), mRNA sequences indicate a second isoform may exist: it is missing the amino acids (aa) 1–78 (the cytoplasmic domain, the transmembrane anchor protein, and 17 aa of the extracellular domain). This isoform would likely be expressed intracellularly as MARCO is a type II transmembrane protein, and thus only appear in body fluids after membrane rupture (36). Whether MARCO could be shedded from the membrane is unclear. MARCO is primarily expressed on alveolar macrophages in the lung and is important for clearance of exogenous material after inhalation. It has been shown to play a critical role in development of silicosis in a mouse model (23). Our finding that MARCO binds to various naked, endogenous crystals like MSU, t-CPPD, cholesterol, and calcium oxalate dihydrate in addition to exogenous, inhaled particles (e.g., silica) shows that it might act as a more general receptor for many crystalline structures. It remains to be seen, if immune cells like macrophages at sites of crystal-induced inflammation outside of the lung express MARCO and whether it plays a non-redundant role as in the lung. As a phagocytic receptor MARCO could potentially enhance phagocytosis of crystalline structures. This could be beneficial if the crystals are then digested by the macrophage but detrimental if the ingested crystals enhance production of inflammatory cytokines and/or activation of the inflammasome. MARCOs ability to recognize so many distinct crystalline surfaces might be due to the formation of homo- or hetero-oligomers as described for the scavenger receptor SR-B1 (37, 38). A multimerization in a crystal-like flat surface leading to high avidity and increased binding to various crystals based on the flat surface may be conceivable.

The circulating ectodomain of transmembrane LDLR has first been discovered as an interferon-induced antiviral protein (39, 40) and has recently been described as a possible novel marker for inflammation (41). Thus, an association of this soluble LDLR to crystals—as demonstrated by LC-MS—also appears reasonable. We show that recombinant LDLR binds to most opsonized crystals and that this binding correlates with the binding of LDL: both LDL and LDLR bind to all crystalline structures tested, but not cholesterol crystals. We also observed binding of LDLR to some unopsonized crystals, so we cannot exclude that it may also act as a receptor for pure particles.

Binding of LDL and LDLR to crystals was conserved between human and mouse. However, we did not see major alterations in phagocytosis or IL-1β production in response to crystals by mouse immune cells deficient for LDLR. Since we expect that most of the crystal surface is covered with LDL in the presence of serum, it is conceivable that either the cells have other recognition mechanisms for coated LDL or for different opsonizing proteins like complement. Other receptors could potentially compensate the loss of LDLR: members of the LDLR family, e.g., LDLR-related proteins (LRP) that are expressed in a number of different tissues with a wide range of different ligands, could be able to bind LDL coated on crystals and induce endocytosis of crystals. Alternatively, a recent study suggests that direct interaction of a solid structure with the cell membrane can lead to receptor-independent phagocytosis (42). It would be interesting to see, if LDL or other crystal-coating proteins block this direct interaction with the membrane.

LDL, the major cholesterol-carrying lipoprotein of plasma, transports lipids from the liver to peripheral tissues, where it is mainly taken up by binding to the membrane-bound LDLR. LDL is believed to be the main culprit in the development of atherosclerosis. However, it is unclear if elevated LDL is sufficient to trigger plaque formation or if it only binds to the damaged artery wall (43). Potentially, LDL may actually act as a patch to cover damaged surfaces and only the prolonged and excessive plaque formation leads to a detrimental outcome of an originally beneficial mechanism. Previous data (19, 20) as well as our own data demonstrate that LDL shows remarkably strong binding to various crystalline structures. LDL may even be depleted from serum by addition of MSU crystals (data not shown). Such strong and specific interactions would support the notion that LDL acts as a patch on damaged or unnatural surfaces. We further show that LDL opsonization inhibits the production of ROS induced by various crystalline structures in human neutrophils. Probably, LDL-coating blocks the interaction of crystals with immune receptors like Mac-1 (CD11b/CD18), Fc-receptors, or the membrane itself (44).

One puzzling finding that we cannot explain is the fact that LDL strongly inhibits cholesterol crystal-induced ROS production while we were unable to show any binding of LDL to cholesterol crystals. Maybe this interaction is too weak or transient to be observed with the methods used, or cholesterol crystals induce ROS by a distinct pathway that does not rely on the interaction with the crystal surface, e.g., release of soluble cholesterol that interacts with the cells.

Furthermore, our findings indicate that LDL reduces IL-1β production of PBMCs in response to some crystals. However, this alteration correlates only partly with the reduced ROS production. For rather small MSU crystals (lot2) and silica crystals, we see a strong reduction in ROS but not in IL-1β production. In some experiments, we even saw increased IL-1β production. It appears reasonable to speculate that the determining factor for this phenomenon might be the size of the particles as this specific lot of MSU crystals and the silica crystals were very small. Thus, LDL may inhibit IL-1β production by crystals that are too large to be phagocytosed. Smaller crystals that may be phagocytosed with or without the need for LDL binding receptors may activate other pathways. It is possible that LDL inhibits IL-1β production by preventing interaction with ROS-inducing receptors or direct interaction with the membrane (45), but LDL may be unable to inhibit phagosomal destabilization after phagocytosis, which would activate the inflammasome (46). Alternatively, other activating receptors may only be active in the endocytic compartment. CD36 recognizes oxidized LDL and activates TLR signaling only after endocytosis (47). Since the LDL used in our experiment could get oxidized during the incubation period, small crystals could deliver the oxidized LDL to CD36 in the endocytic compartment while large crystals could not. It remains to be seen, if the observed effects of LDL-coating on IL-1β production are actually caused by priming, i.e., pro-IL-1β production or inflammasome activation itself. LDL may also inhibit post-transcriptional regulation of IL-1β mRNA, which has been shown to be important in IL-1β production by MSU activated monocytes (48).

Oxidization or other modifications of LDL may alter both its binding to crystals and its effect on immune cell activation. It is also unclear if LDL coating effects other crystal-induced responses like complement activation and coagulation. Future studies are thus required to understand a possible role of LDL in crystallopathies.

Together, our unbiased LC-MS approach has identified one receptor that directly recognizes disease-associated crystals (MARCO) and with LDLR another receptor that binds to all crystals tested, except cholesterol, when they are opsonized with LDL. We further show that LDL not only binds to MSU crystals but all crystals tested, except for cholesterol crystals or microbes like fungi. This LDL binding leads to strong inhibition of ROS production but variable effects on IL-1β production depending on crystal sizes.

There is a large range of crystal-associated pathologies which makes it important to investigate if there is a shared molecular basis between the recognition of different crystals. By describing the interaction of disease-associated crystals with MARCO, LDLR, and LDL we have found novel and unexpectedly specific interactions of proteins with crystalline structures. We hope these data will spur future research into the functional role of these three proteins in different crystallopathies.

## Data Availability Statement

The raw data supporting the conclusions of this article will be made available by the authors, without undue reservation.

## Ethics Statement

The studies involving human participants were reviewed and approved by the Ethics Committee of Hannover Medical School (Ref. No: 3395-2016). The patients/participants provided their written informed consent to participate in this study.

## Author Contributions

KN supervised the research. AA and KN planned experiments. AA, AK, LH, and AW performed experiments. AP performed LC-MS analysis. AA and KN wrote the manuscript. All authors contributed to the article and approved the submitted version.

## Funding

This work was funded by a grant from Deutsche Forschungsgemeinschaft (DFG) to KN (grant number: NE 2206/1-1).

## Conflict of Interest

The authors declare that the research was conducted in the absence of any commercial or financial relationships that could be construed as a potential conflict of interest.
