# Supplementary material for: Binding of Macrophage Receptor MARCO, LDL, and LDLR to Disease-Associated Crystalline Structures
Source: Front Immunol. 2020 Dec 8;11:596103. doi: 10.3389/fimmu.2020.596103 (PMC7753766; doi:10.3389/fimmu.2020.596103)
Supplement: Supplementary file 1 [file DataSheet_1.pdf]

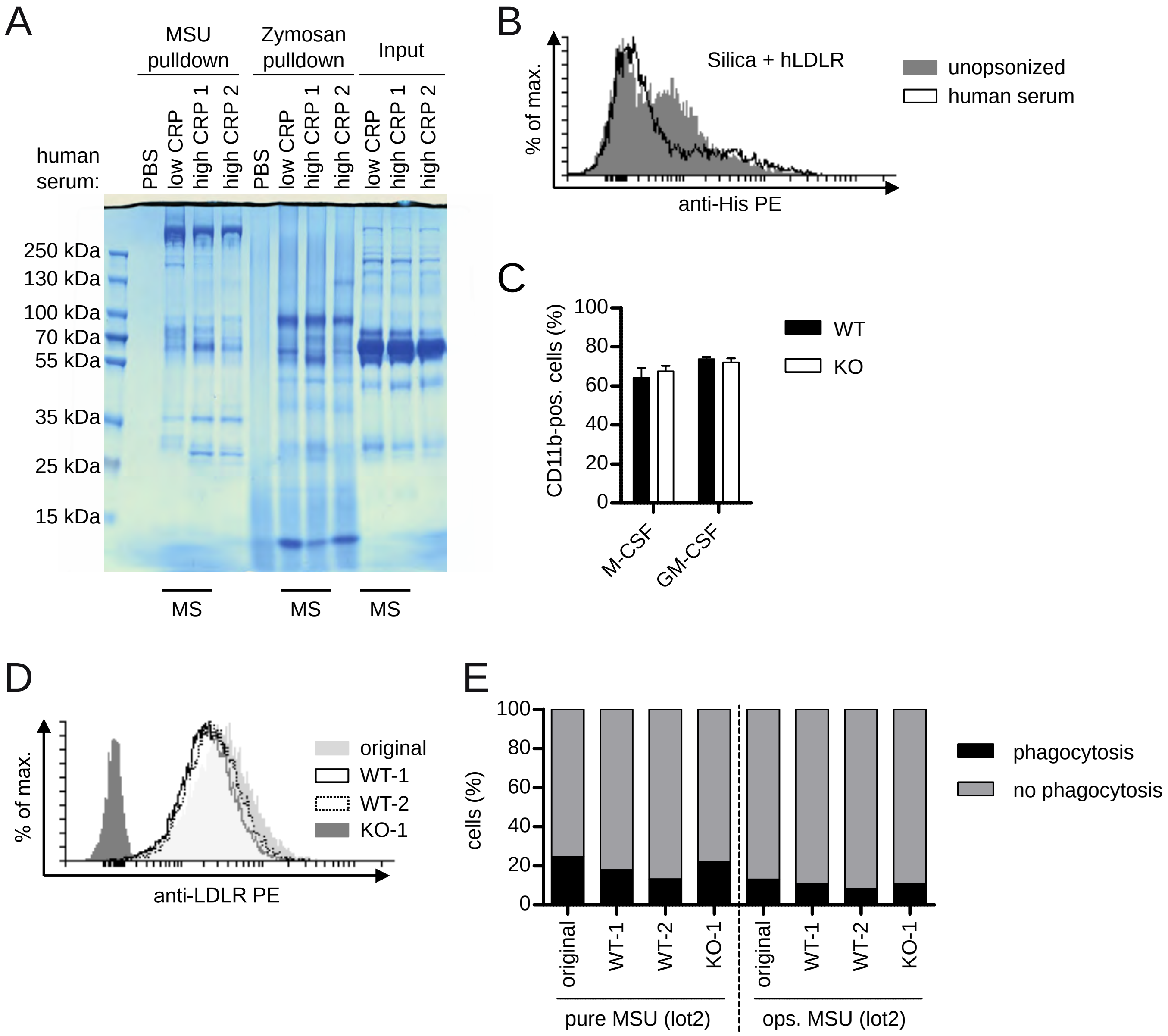

Supplementary Figure 1.
(A) Uncropped SDS gel underlying the LS-MS analysis results in Figure 1A. **(B)** Binding of hLDLR to unopsonized (HBSS) and opsonized (human serum) silica crystals; histograms depicting the results of flow cytometry analysis in Figure 2B are shown. **(C)** CD11b expression of M-CSF- and GM-CSF-differentiated murine cells used in Figure 4. **(D)** LDLR expression on HepG2 cells: untreated cells (original) and CRISPR/Cas9-treated clones (WT-1, WT-2, and KO-1) were stained with anti-hLDLR PE. **(E)** HepG2 cell clones (see **(D)** for LDLR expression) were incubated with MSU (lot2) at 37°C for 30 min. Opsonized MSU crystals (ops.) were incubated with human serum at 37°C for 30 min and washed with HBSS before adding to the cells; unopsonized crystals (pure) were stored in HBSS. The amount of phagocytes was determined by counting the cells with/without intracellular MSU crystals using a polarization microscope with a 20-fold magnification.

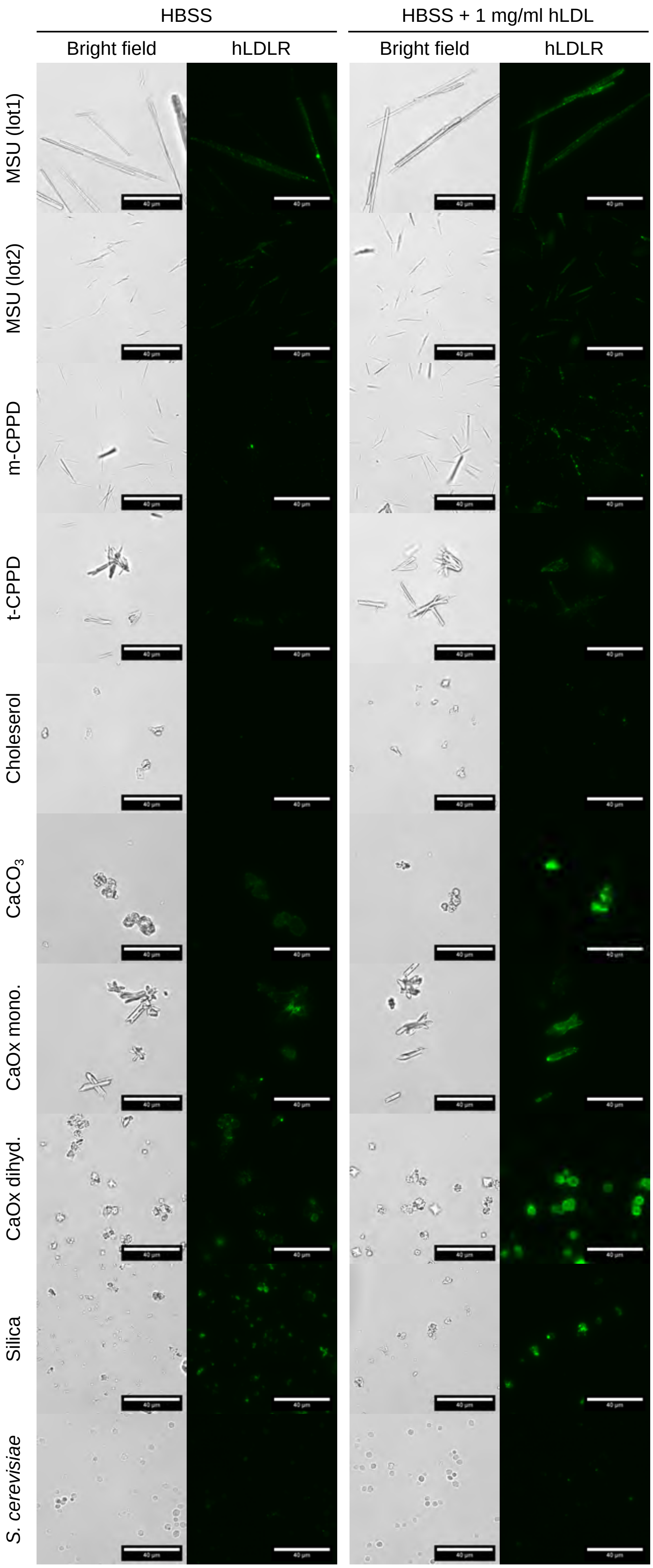

Supplementary Figure 2.
Indicated crystals were opsonized in HBSS or HBSS containing 1 mg/ml human LDL at 37°C for 30 min. After washing with HBSS the particles were incubated with 5 µg/ml recombinant His-tagged protein in HBSS + 5% BSA at 4°C for 60 min (hLDLR). Protein binding was analyzed using mouse anti-His Tag plus goat anti-mouse AlexaFluor488. Fluorescence of the samples was detected by fluorescent microscopy; scale bar = 40 µm.

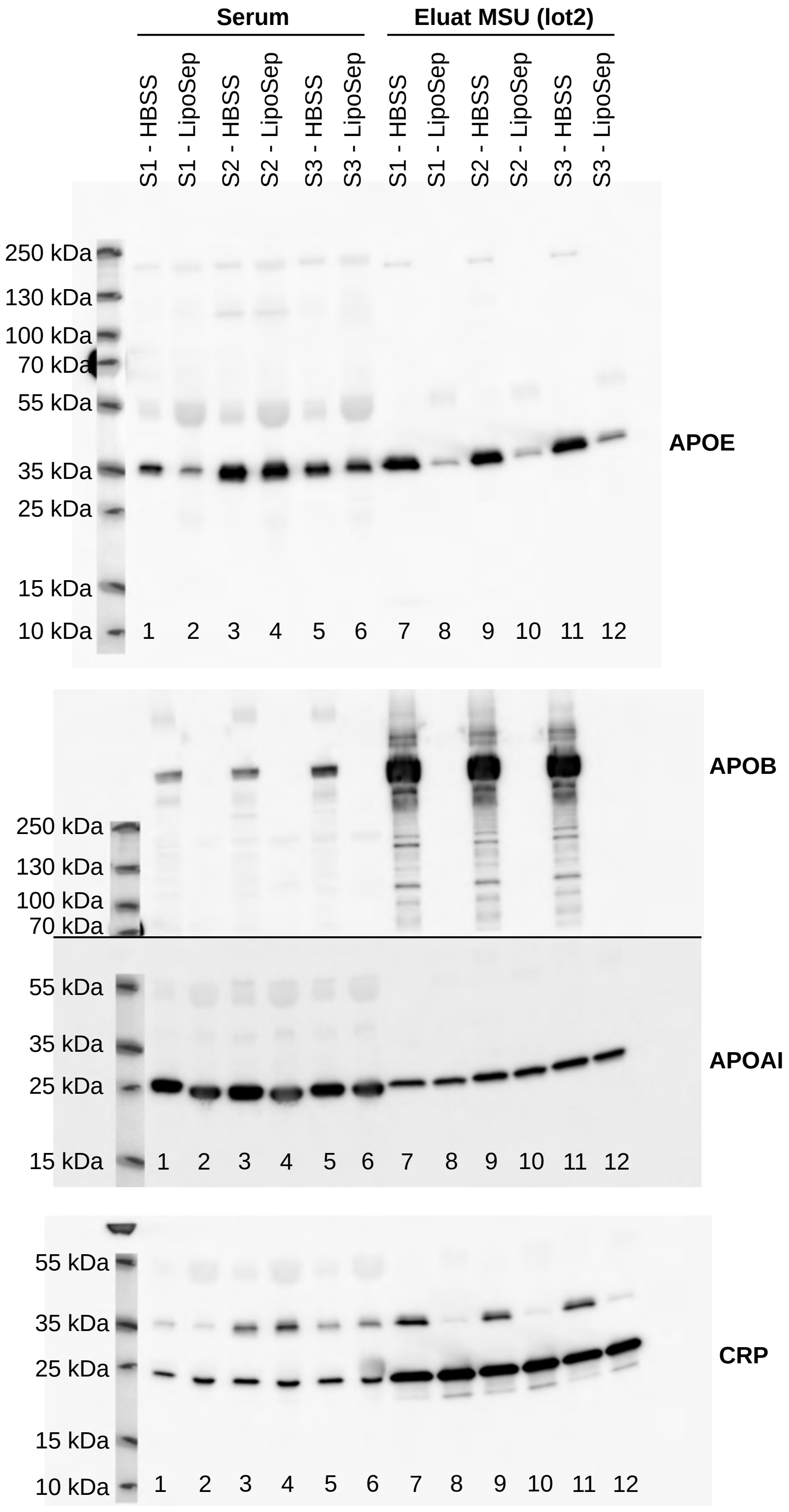

Supplementary Figure 3.
Sera from three individual healthy donors (S1-3) were treated either with HBSS (negative control) or LipoSep Immunoprecipitation Reagent (LDL-depleted serum). These sera were directly analyzed (serum) or incubated with MSU (lot2) crystals and crystal binding proteins were eluted and subjected to Western blot analysis. Uncropped anti-ApoE, anti-ApoB, anti-ApoAI and anti-CRP Western blots are shown.

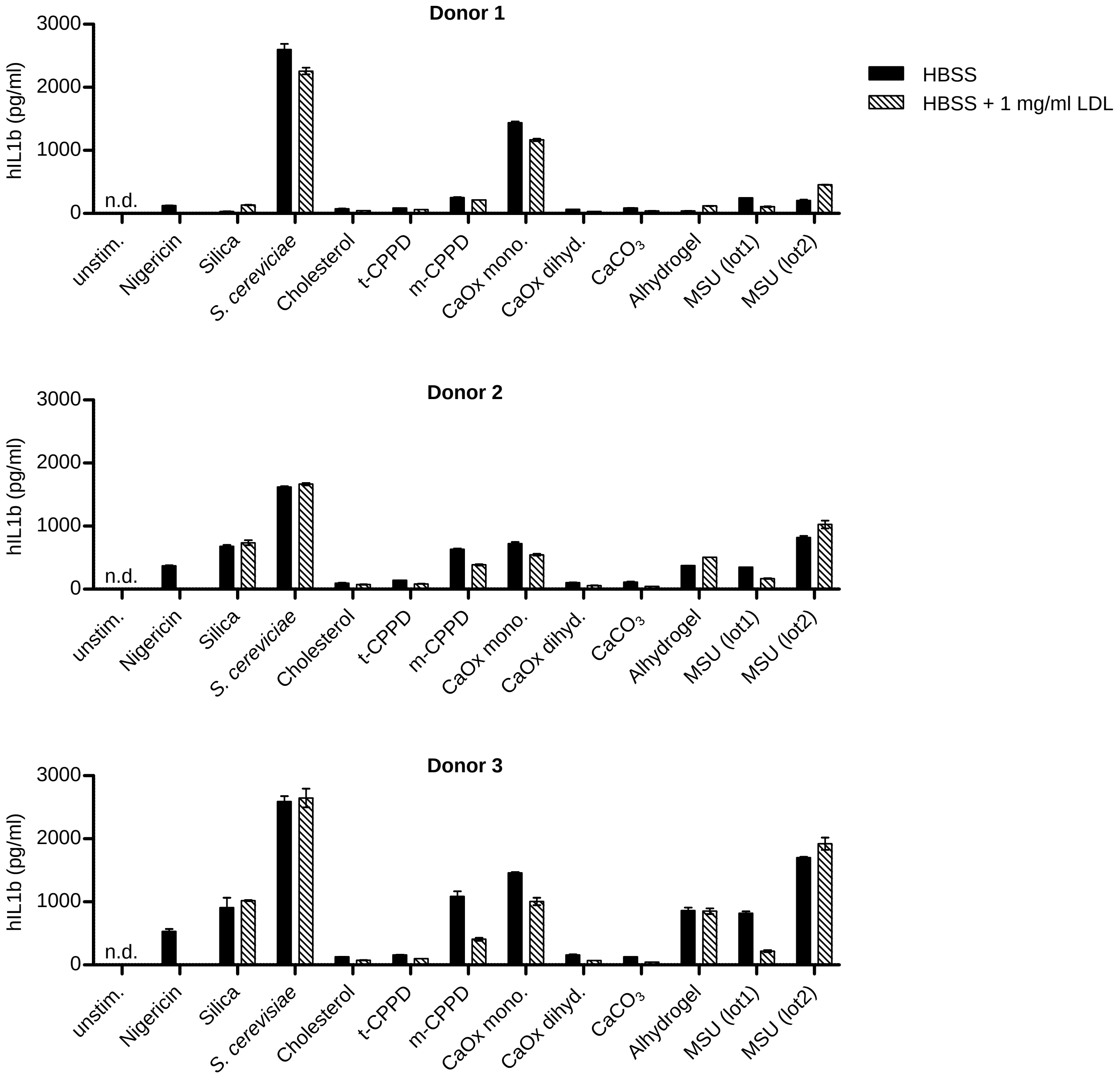

Supplementary Figure 4.
Human peripheral blood mononuclear cells (PBMCs) of three independent donors were stimulated with the same unopsonized and opsonized particles used in Figure 5A. Supernatants were harvested after 16 h of incubation at 37°C and subjected to ELISA. The IL-1β production of all three donors (1 - 3) is depicted.

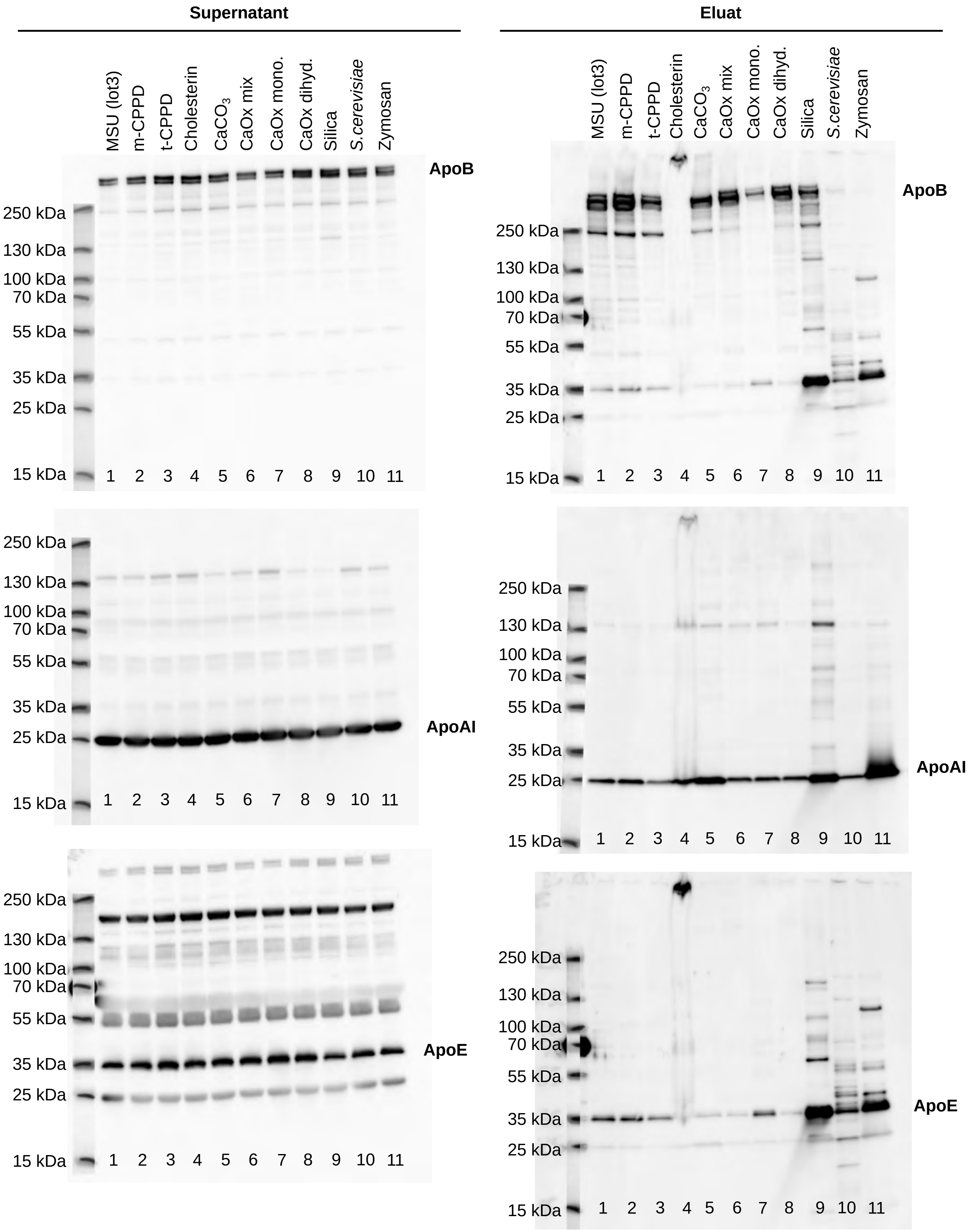

Supplementary Figure 5.
Uncropped anti-ApoB, anti-ApoAI, and anti-ApoE Western blots from Figure 3D.

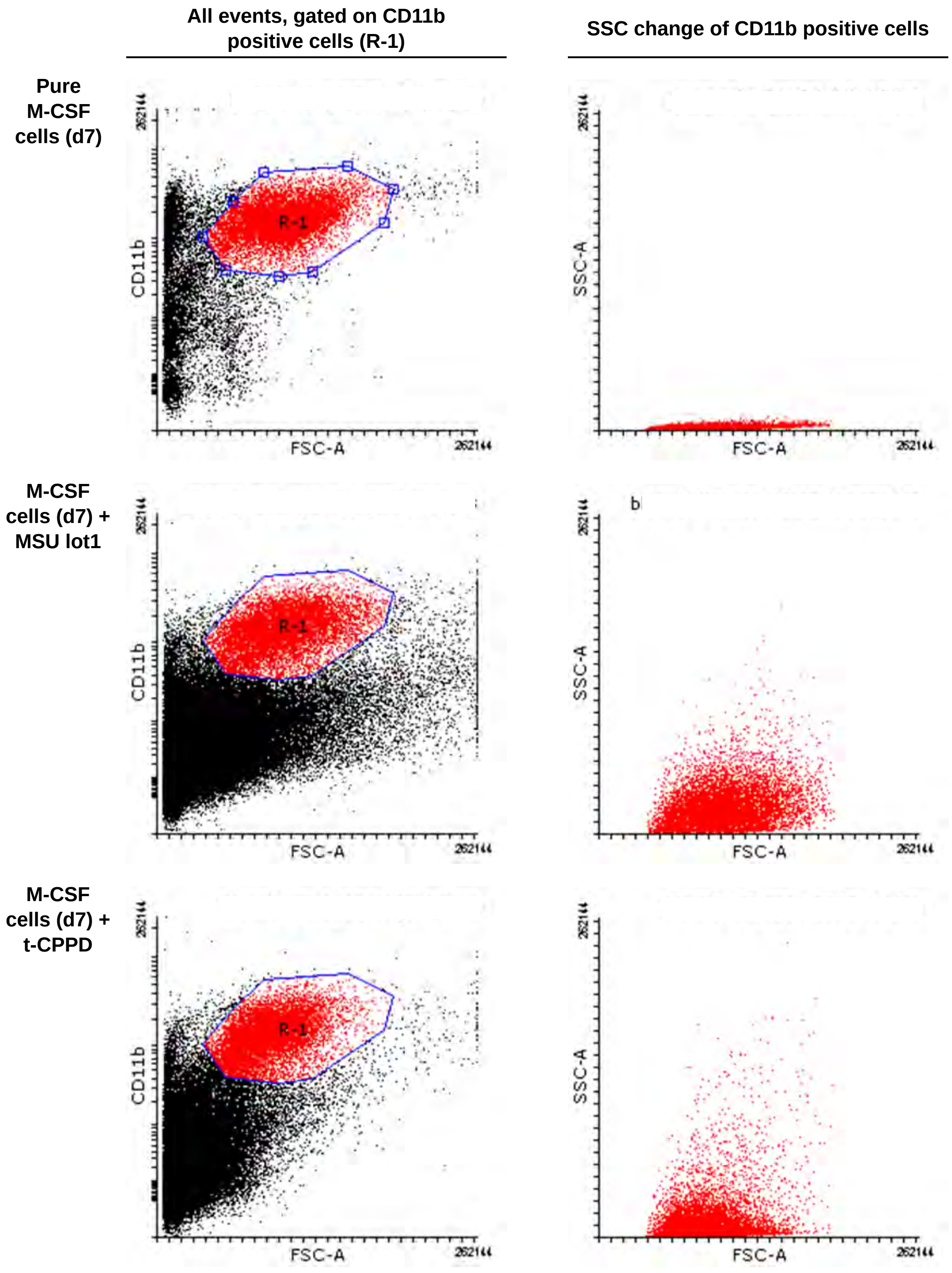

Supplementary Figure 6.
Representative gating strategy for phagocytosis analysis of GM-CSF- and M-CSF-differentiated cells in Figure 4A. Using flow cytometry and flowing software all events were measured and a gate was set on the CD11b positive cells (R-1) to distinguish cells from crystals (left). Then, the increase in cellular granularity (sideward-scatter, SSC) of these CD11b positive cells was assesed (right).
